# Testing a Calibration - Free Eye Tracker Prototype at the Kunsthistorisches Museum in Vienna

**DOI:** 10.16910/jemr.13.2.10

**Published:** 2020-11-10

**Authors:** Zoya Dare, Hanna Brinkmann, Raphael Rosenberg

**Affiliations:** Department of Art History, University of Vienna, Austria; Department of Arts and Cultural Studies, Danube University Krems, Austria; Department of Art History and Vienna Cognitive Science Hub, University of Vienna, Austria; MECS, University of Lüneburg, Germany

**Keywords:** eye tracking in museums, art, paintings, viewing time

## Abstract

Eye tracking research in art viewership is often conducted in a laboratory setting where reproductions must be used in place of original art works and the viewing environment is less natural than in a museum. Recent technological developments have made museum studies possible but head-mounted eye tracking gear and interruptions by researchers still influence the experience of the viewer. In order to find a more ecologically valid way of recording eye movements while viewing artworks, we employed a prototype of a calibrationfree remote eye tracker hidden below selected paintings at the Kunsthistorisches Museum in Vienna. Museum visitors were unaware of the study and informed post hoc that we had registered their viewing behavior and asked to give consent for the use of their data. This article presents the study design as well as results from over 800 participants. While the data quality from the eye tracker prototype was not sufficient to conduct the intended analysis on within-painting gaze movements, this study might serve as a step towards an unobtrusive examination of the art viewing experience. It was possible to analyze time spent viewing paintings and those results show that certain paintings consistently drew significantly more prolonged attention from viewers.

## Introduction

Eye tracking is a particularly relevant method for the investigation of art reception as the eye is crucial in our interaction with visual artworks. The typical encounter between the viewer and an artwork occurs in a museum. However, until recently, eye tracking investigations of the perception of artworks were only possible in laboratory settings, using reproductions of artworks that are most often viewed on a screen. While producing precise data, the lab setting is a far cry from the natural museum environment: the artworks are not original; the context with its specific relations between the space, the visitors, and the artworks is missing entirely; the participants are expected to sit in a chair with restricted mobility; and finally, the device must be calibrated, meaning that participants are aware that their experience is being watched and scrutinized. 

Due to these limitations, researchers have attempted various methods of moving into the more natural viewing environment of museums. Bachta et al. (1) brought the lab into the museum by setting up their remote eye tracker in front of original paintings instead of reproductions, and numerous groups have employed mobile eye tracking, using wearable equipment to allow their participants some degree of mobility (2, 3, 4, 5, 6, 7). However, mobile eye tracking equipment still needs to be calibrated, and the viewing experience is disturbed by the presence of eye tracking headsets and researchers. Participants wearing headsets in the gallery might attract the attention of other visitors and therefore feel further observed during their experience. Furthermore, previous studies have been conducted on a limited scale with only a handful of participants due to equipment and set-up restrictions. 

Visitor observation studies, another common method of studying art viewing in museums (8, 9, 10), offer solutions to some of these issues but also present some of their own. While in some cases visitors can be observed without the explicit knowledge that they are being watched, the experimenter cannot always tell where the participant is looking and the time estimates are less precise. For example, in Smith and Smith (2001) a visit of less than 3 seconds was not recorded, while they themselves admit that it is most often that visitors pass by paintings quickly. A lot of visual information can be gathered by the brain in three seconds of viewing but those glances are nearly impossible to study without an eye tracker.

This study, conducted at the Kunsthistorisches Museum in Vienna, Austria, aimed to combine the unobtrusive nature of visitor observation studies with the precision of eye tracking by employing a calibration-free, concealed “gaze tracker” prototype. With the help of this device, we were able to gather data from museum visitors who became aware of the recording only after the data had been gathered, at which point they were asked to consent to the use of that data. This approach allowed for the recording of a greater number of participants (N=808) than previously possible, as they did not need to be fitted with equipment and individually calibrated. The device allowed for the recording of the most natural viewing experience thus far in a museum context.

The primary goal of the study was twofold: (1) to compare viewing time of participants under these less obtrusive conditions with past studies and (2) to conduct more in-depth analysis on where participants looked within each painting, looking for any variation based on visitor demographics, namely gender and culture. However, the calibration-free eye tracker ultimately failed to produce data accurate enough to compare viewing patterns within the single paintings. However, it is possible to determine whether the gaze was directed at the painting and analyze the duration participants viewed each work. We report here these results and the innovative study design that might be of interest for future studies when developments in eye tracking technology will improve the accuracy of similar devices.

## Methods

### Apparatus

**Figure 1.**
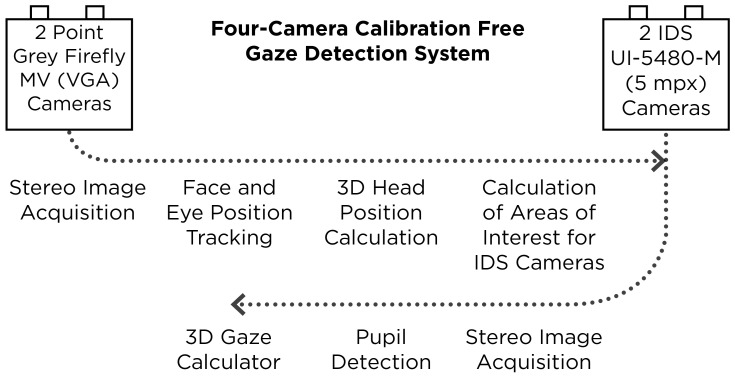
A diagram showing the function of the calibration free gaze tracker.

**Figure 2.**
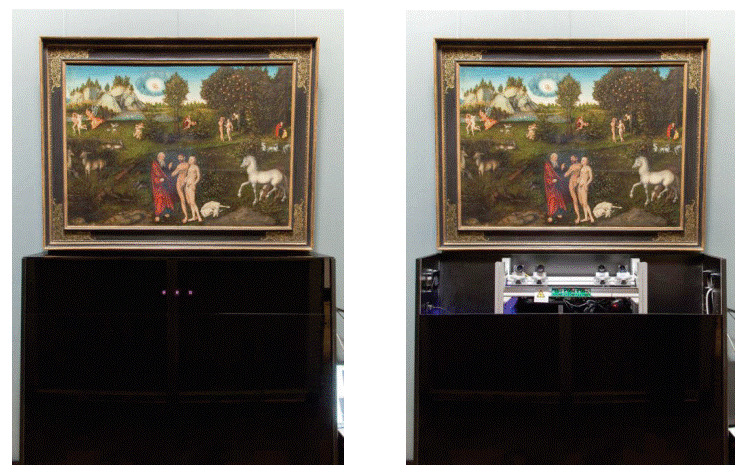
A view of the painting as seen through the participant's viewing window. The concealed eye tracker was placed under the painting and covered by plexiglass (left). Inside, four cameras monitored the viewing window (right). Lucas Cranach, Paradise, 1529

A calibration procedure enables the linkage of pupil position with the precise point seen at a given distance by the test person. Since a machine can detect the optical axis running between the pupil and the center of the eyeball and since the fovea centralis is located approximately at the back of this axis, it is theoretically possible to build an eye tracker that would not require any calibration. Such a calibration free “gaze tracker” was designed and mainly used in vehicles where the head is more or less stable; two cameras are used to locate and track the eyes (11). Our prototype was created by the Institute for Bio-Inspired Computing of the Fraunhofer Institute for Digital Media Technology in Ilmenau (Germany) and adapted for the museum setting in order to allow for more distance between the equipment and participant and a larger headbox. In our setup, the head was able to move more freely and additional cameras were used to first detect the face, and then the eyes. The system consisted of a structure made of aluminum rails, a PC, two WiFi antennas, two five-mega-pixel IDS gaze tracker cameras, two infrared VGA Point Grey face tracker cameras, a power distributor, a board with infrared LEDs and cabling. The structure’s dimensions were 70cmx50cmx20cm (x, y, z axes). The cameras and infrared lighting were mounted on two cross rails (Fig. 1 and 2). We tested this prototype for the first time in this altered setup.

This construction was contained in a simple box made of veneered plywood with two shelves. It did not stand directly on a shelf but was fixed with screws to the back wall of the box and could therefore be easily adjusted. The front and top of the box were covered with black, infrared-permeable Plexiglas sheets. The system could be directly accessed through the on-board PC, or wirelessly through the specifically designed gaze tracker app on a mobile phone running Android 4.0.1 or higher. The app function will be discussed further in the Procedure section below.

The calculation of the gaze vectors is based on the detection and calculation of the pupil ellipse. The pupil ellipse makes it possible to calculate the optical axis with the help of a Hough Transformation. In addition, models are built into the software to compensate for individual deviations between the optical axis and the visual axis ( 12, 11).

**Figure 3.**
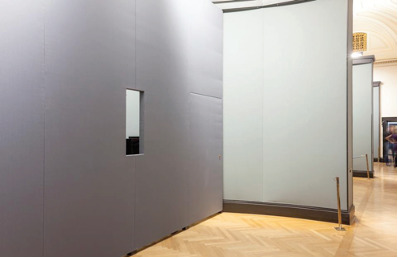
The viewing window created to place the participants inside the recording headbox.

The device recorded the position of the pupil at a resolution of 100 Hz inside a predefined headbox. This means that viewers had to be at the right distance from the eye-tracker and the right position, standing in the middle of the cameras. To ensure this, walls were built in front of the paintings to be recorded. In order to achieve an inconspicuous appearance, the walls were covered with a fabric that matched the surrounding exhibition rooms of the Kunsthistorisches Museum. A 70 cm high "window" was cut into this wall in order to bring the visitors to position their heads in the right place for recording (Fig. 3). While this did alter the previous museum space, most participants reported assuming that the walls were set up for conservation purposes of a particularly fragile work. The head box of the gaze tracker was 25 cm in height and 35 cm in width at given distances: Depending on the size of the paintings, one device was set at a distance of 130 cm (for the smaller paintings), the other at a distance of 150 cm (for the larger paintings).

### Stimuli

The study was conducted for four weeks, in September 2014 at the Kunsthistorisches Museum Vienna. We used two gaze trackers and hence tested two paintings at a time. Depending on the canvas size, the paintings were displayed above one of the two systems at either a 130 or 150 centimeters distance from the window. The first two paintings were displayed for two weeks; the first week was set aside for testing and only a few participants were recorded that week. We tested a total of six paintings with the display changing every week on Mondays, when the museum was closed. The experiment ran for three weeks, preceded by a test week when only a few participants were recorded. For a list of the paintings see Table 1.

**Table 1:** List of paintings used for the study including dimensions and distance from the viewing window.

**Artist**	**Title**	**Year**	**Dimensions (height x width in cm)**	**Week Displayed**	**Distance (cm)**	**# of views**
Pieter Aertsen	Vanitas Still Life	1552	61.5 × 101	Week 0&1	130	178
Lucas Cranach	Paradise	1529	81 × 114	Week 0&1	150	202
Johannes Tilens	Mountain Landscape	c. 1610	62 × 94	Week 2	130	175
Titian	Mars, Venus and Cupid	1488	97 × 109	Week 2	150	133
Andrea Mantegna	St. Sebastian	1475	68 × 30	Week 3	130	117
Pieter Coecke van Aelst	The Rest on the Flight into Egypt	c. 1530-40	112 × 70.5	Week 3	150	123

### Procedure

**Figure 4.**
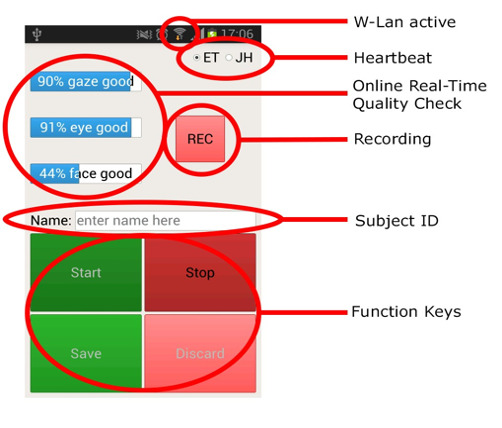
A screenshot of the mobile phone application interface. It allowed the researchers to remotely start and stop the recording process, save or discard the recorded data based on participant consent, enter the participant ID, check the recording quality, and ensure continuous connection to the gaze tracker through the W-Lan and “Heartbeat” signal from the gaze tracker (ET) and the job handler (JH).

At the entrance to the Kunsthistorisches Museum signs were posted letting visitors know that filming (with no mention of eye tracking) was being conducted for research purposes. The experiment was set up in a part of the gallery (Kabinett 19) which is laid out as a long hallway with alcoves on one side (and closed windows on the other) normally containing three paintings each. The two devices were set up at the opposite two sides of the hall, each in one of these alcoves. Visitors entering the gallery proceeded at their own pace to the viewing windows. When they approached a window, the researcher—across the room and dressed as museum staff—would activate the eye tracker using the app on a smartphone. The app allowed the researcher to enter a participant number and do a live data check of face and eye detection (Fig. 4). The app was used to start the recording process and turn it off when the visitor stepped away from the headbox. Once the visitor moved on to the next room, they would be approached by the researcher, told of the study, and asked for consent to use their data. At that point, a consent form would either be signed or the data would be deleted immediately. The data could be saved or deleted directly from the mobile app. If consenting to participate, the visitor would then fill out an extensive questionnaire set up to analyse the general demographic, the gender identification, the sexual orientation, the cultural background and the art-expertise as possible influencing factors for the viewing behavior.

### Participants

**Figure 5.**
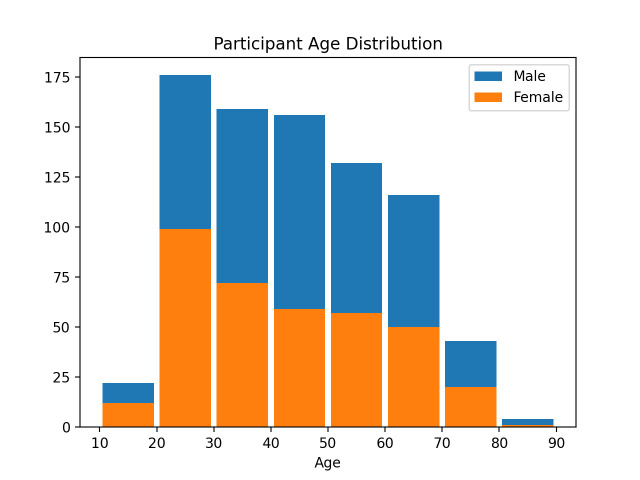
Distribution of participants by age and sex. Total number of participants is 808.

All of the participants were independent museum visitors, not brought to the museum especially for the study. Over the course of four weeks, 808 participants over 18 years of age took part in the study (54.86% male, sd=15.93). Nearly half were visiting the museum for the first time. A questionnaire was available in German, English and Japanese. A breakdown of age and sex can be seen in Figure 5. The majority of participants, 77%, had some higher education and 72% had no knowledge of the paintings in the study prior to their visit.

## Results

As mentioned above, only viewing duration results can be reported. A more detailed analysis was planned (see Introduction), but since the expected accuracy of the calibration-free gaze tracker was not achieved, this further analysis was not possible. While data quality was not good enough for a consideration of which elements of the painting participants looked, it was possible to distinguish glances on and off the paintings at large.

**Figure 6.**
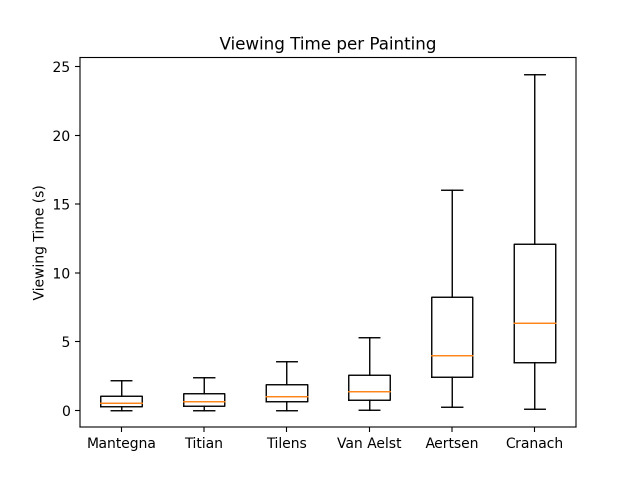
Total time spent viewing each painting.

The results of a one-way ANOVA and follow up t-test comparisons revealed that the viewing time for single paintings significantly differed: *Paradise* by Lucas Cranach gathered the longest views (mean 10.5 seconds, median 6.4 seconds), followed by *Vanitas Still Life* by Pieter Aertsen (mean 6.1 seconds, median 3.9 seconds). *St. Sebastian* by Andrea Mantegna received the least interest (mean 0.8 seconds, median 0.5 seconds; Fig: 6). The difference between the two longest viewed works and the other paintings is highly significant (p< 0.001, d=0.9). The average time spent in front of the head box for all the paintings was 11.5 seconds with the longest time registering at 138 seconds. However, when considering time spent looking at the painting directly, the average viewing time dropped to 4.3 seconds with the longest time being 126 seconds. The remaining time was spent looking at the walls and other architectural features of the space behind the window and will not be counted in the following analysis.

Out of 924 instances of viewing (some visitors viewed the paintings in both gaze tracker systems during their visit) only four spent over a minute looking at any painting. These few participants, however, influence the average and it must be noted that the general median viewing time was only 1.74 seconds.

**Figure 7.**
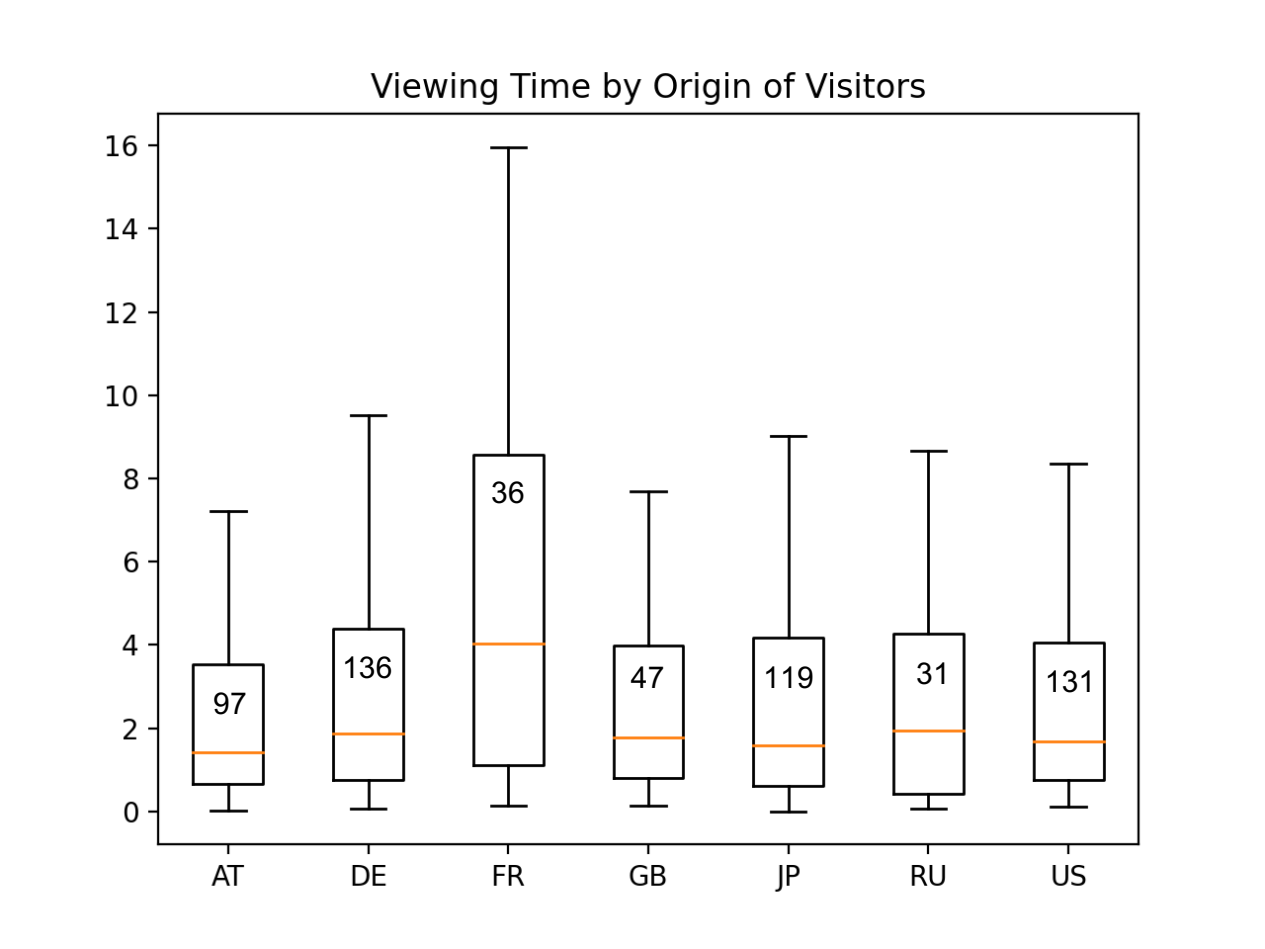
Time each nationality spent viewing the painting. The French viewed paintings for significantly longer than participants from other nations.

One of the goals of the study was to include visitors’ different backgrounds, such as culture and gender, into the analysis and look for viewing behavioral differences between groups. The study included participants from 60 different countries. The top seven (with 30 participants or more) were, in descending order: Germany (DE), Japan (JP), USA (US), Austria (AT), Great Britain (GB), Russia (RU) and France (FR). These seven countries represent 65% of all participants. An ANOVA and follow-up t-tests revealed that only one group is significantly different from the others: the French viewed the paintings significantly (p<0.0008, d=0.6) longer than others (Fig. 7).

While men viewed the paintings on average for a slightly longer time, the longest recorded time was that of a female participant. Both the difference between the gender and according to sexual orientation (divided into five groups: heterosexual, homosexual, bisexual, asexual, and other) of the participants is not statistically significant.

## Discussion

The experiment’s design was successful in eliminating many of the usual interruptions that occur in a viewer’s museum experience. There were no calibration, visible equipment, or interaction with researchers until after the recording took place. This meant that participants did not know they were being tested, providing us with the opportunity to record natural viewing experiences. The changes we had to make in the museum display did not interrupt the visitor’s experience of the works, they did allow data recording, but the system did not deliver accurate results. However, once improved, the device could be used to analyze not only viewing duration but within-painting gaze paths and events within specific areas of interest. These can, in turn, shed light on elements of the paintings that might capture viewers’ attention and be compared to the data taken in a lab setting in order to better understand whether there is a difference in perceiving works in a museum as opposed to a laboratory. Such data will open new horizons both for the study of the reception of single paintings by different audiences and our understanding of museum visitors’ viewing experience more generally.

This approach allowed us to test over 800 participants in less than a month, which is vastly more than any study has been able to do in a museum context with original artworks. For comparison purposes: In 2018 and 2019, our lab conducted another large-scale museum study, this time with mobile eye tracking headsets (7). Using a slightly higher number of team members and four eye tracking devices at a time, we were only able to test up to 150 participants in seven days.

A remarkable result of this study is that the paintings that were exhibited at the same spot in the museum and viewed by a similar amount of visitors, received significantly different viewing times, varying from a median of 6.4 to 0.5 seconds. We later had similar results in the already mentioned study conducted with a mobile eye tracker at the Belvedere Museum in Vienna: certain artworks received significantly different viewing times varying from a median of 0.52 to 47 seconds, and it is noteworthy that the same artworks attracted similar viewing times in different display situations (7). Smith and Smith (8) also found differences between time spent in front of the paintings they tested. They attributed the variations in viewing time to size of canvas, fame, and available seating in front of the work. These factors do not apply to the current study since none of our paintings are popular highlights of the museum, they had similar sizes and were displayed in the same place.

The difference in viewing time suggests that there is something about certain artworks themselves that consistently draws more attention from a wide variety of viewers. Systematic studies with a larger number of paintings as well as within painting viewing analysis may shed light on which elements attract longer visitors’ attention.

The viewing duration obtained in our study are lower than previously reported viewing averages. For instance, Smith and Smith ( 8) observed an average of 27.2 seconds at the Metropolitan Museum of Art in New York, with a median of 17 seconds. However, their results are not directly comparable, since the artworks used for their study were highlights of the museum which would likely have been known at least by some visitors in advance of their visit. This was not the case for the paintings used here. Mantegna’s *St. Sebastian*, arguably the best known among our paintings, was the one with the lowest average viewing time. It must also be noted that we were able to separate the time spent viewing a painting from just standing in front of it. Earlier visitor observation studies that did not use an eye tracker could not know exactly where the participant was looking and would therefore record the total time spent in front of a painting, whether looking directly at it or not.

The comparison in viewing time revealed no significant difference between sexes or persons with different sexual orientations. Notably, this applies regardless of the content of the paintings. One of them showed a female nude (Titian, *Mars and Venus*), another a nearly naked man (Mantegna, *Saint Sebastian*), but neither of those caused an increase of viewing times for any group. In regard to cultural differences, the only relevant result that we can report (and cannot explain at the moment) is that participants born in France had a significantly longer viewing time.

Beyond the technical problems, our innovative method is, of course, not without limitations. The museum display still had to be altered to record data and did not allow for visitors to approach the paintings as close as they could for other works in the museum: The wall with the window altered the normal viewing situation within the museum, though this also occurs in museums where similar measures are used to create distance between visitors and artworks. Another limitation of the window set up was that it did not work if visitors looked through the window from afar (in which case they would not have been able to see the whole painting) or took a photograph (this would result in blocking their faces and arguably should not count as beholding time). While it did allow for the testing of a large number of participants, there also needed to be a device for every painting—which makes it costly to test a large number of paintings. As eye tracking hardware and algorithms develop, we assume that it will be possible to use similar devices for a more in-depth analysis of museum viewing than has yet been possible.

## Ethics and Conflict of Interest

The authors declare that the contents of the article are in agreement with the ethics described in http://biblio.unibe.ch/portale/elibrary/BOP/jemr/ethics.html and that there is no conflict of interest regarding the publication of this paper. The ethics commission of the University of Vienna approved this study on 29-04-2014 (00060).

## Acknowledgements

We would like to thank the team of the Picture Gallery of the Kunsthistorisches Museum Vienna for their unceasing dedication in helping to conduct this study, in particular the then Director Sylvia Ferino and Bertrun Kos as well as Peter Gregorc, Barbara Herbst, Verena Hofer, Elke Oberthaler, Elvir Osmanovic, Kurt Schopfhauser, Monika Strolz, Christine Surtmann, and Elisabeth Wolfik. We thank all the colleagues from the lab who helped to conduct the study: Mario Thalwitzer, Jane Boddy, Luisa Brigato, Anna Burgstaller, Julia Häussler, Tanja Jenni, Chiara Pompermaier, Nora Rosenberg, and Jasmin Rückert. We thank Hideyuki Hoshi and Maja Kapitler for their help to process the data, Judith Herunter for proofreading, and Helmut Leder for his advice. This research was funded by the Austrian Science Fund (FWF grant P25821).

## Apendix

### Paintings used in the study

Pieter Aertsen, Vanitas Still Life, 1552 (https://www.khm.at/objektdb/detail/31/?offset=1&lv=list)

Lucas Cranach, Paradise, 1529 (https://www.khm.at/objektdb/detail/533/?offset=0&lv=list)

Johannes Tilens, Mountain Landscape, 1610 (https://www.khm.at/objektdb/detail/1926/?offset=0&lv=list)

Titian, Mars, Venus and Cupid, 1488 (https://www.khm.at/objektdb/detail/1954/?offset=0&lv=list)

Andrea Mantegna, St. Sebastian, 1475 (https://www.khm.at/objektdb/detail/1155/?offset=0&lv=list)

Pieter Coecke van Aelst, The Rest on the Flight into Egypt, 1530-40 ( https://www.khm.at/objektdb/detail/495/?offset=0&lv=list)
